# Preoperative Assessment for Surgical Accuracy in Maxillofacial Surgery Cases Using Stereolithographic Models

**DOI:** 10.7759/cureus.60233

**Published:** 2024-05-13

**Authors:** Mohamed Sameer, ArunVignesh KR, Krishnakumar Raja, Ananthanarayanan V, Muthalagappan PL

**Affiliations:** 1 Oral and Maxillofacial Surgery, Sri Ramaswamy Memorial (SRM) Dental College and Hospital, Ramapuram, Chennai, IND; 2 Oral and Maxillofacial Surgery, Sri Ramaswamy Memorial (SRM) Dental College, Ramapuram, Chennai, IND

**Keywords:** precision, cad/cam, oral surgery, stereolithography, 3d

## Abstract

Recent advancements in imaging technologies, particularly stereolithography, have transformed medical and surgical practices, including oral and maxillofacial surgery. Utilizing precise three-dimensional (3D) models crafted from virtual representations, these innovations have revolutionized diagnosis, treatment planning, and surgical simulation. In a study conducted at the Department of Oral and Maxillofacial Surgery, Sri Ramaswamy Memorial (SRM) Dental College, Chennai, five patients with complex maxillofacial deformities underwent surgical interventions guided by stereolithographic models. Despite challenges such as fabrication time and cost constraints, the integration of 3D models significantly streamlined preoperative planning, reduced operative time, and facilitated precise surgical execution. Customized implants and pre-bent plates, based on model simulations, enabled conservative surgical approaches and optimal fit and function. The integration of stereolithography with computer-aided design/computer-aided manufacturing (CAD/CAM) software represents a significant advancement in enhancing surgical precision and improving patient outcomes in cranio-maxillofacial surgery.

## Introduction

The utilization of three-dimensional (3D) models for diagnosis and treatment planning within the medical and surgical domains can be traced back to ancient artistic traditions, exemplified notably by the creation of "moulages" crafted from colored and painted wax. However, the laborious and time-intensive nature of their construction posed significant challenges. Recent advancements in imaging technologies have revolutionized this process, particularly through the advent of reproducible models facilitated by innovation-driven approaches [1].

One of the most remarkable techniques in this realm is stereolithography, a form of rapid prototyping that entails the production of precise 3D physical objects using virtual models and computer-aided technologies. In disciplines like oral and maxillofacial surgery and traumatology, stereolithography plays a pivotal role in diagnosing conditions, planning treatments for facial defects, and simulating surgical interventions. These breakthroughs in diagnostic imaging and surgical methodologies have greatly enhanced surgeons' ability to restore both form and function in patients, facilitating successful prosthetic rehabilitation supported by implants [2].

Despite the significant advances made in the field of cranio-maxillofacial surgery, challenges persist, particularly in cases where patients with complex conditions undergo surgical interventions that result in suboptimal outcomes. These challenges are often attributed to factors such as difficulties in intraoperative assessment and poor visualization of intricate skeletal contours such as the orbit, mandibular condyle, and skull base. To address these hurdles, surgeons are increasingly turning to computer-aided design/computer-aided manufacturing (CAD/CAM) software, which enables the precise planning and execution of intricate cranio-maxillofacial procedures. By utilizing CAD/CAM software, surgeons can develop patient-specific surgical plans, design custom implants, and simulate surgical outcomes to ensure optimal results. The adoption of CAD/CAM technology has revolutionized cranio-maxillofacial surgery, providing surgeons with the tools and resources needed to deliver high-quality care to patients with complex conditions [3].

CAD/CAM software is a useful tool for importing two-dimensional computed tomography (CT) data in Digital Imaging and Communications in Medicine (DICOM) format. This software generates accurate 3D representations of both skeletal and soft tissue anatomy. These 3D representations can be leveraged to fabricate stereolithographic models or manipulated through segmentation, reflection, or insertion of specific anatomical regions to aid in treatment planning. This technology is particularly beneficial in the medical field, where the ability to visualize and manipulate anatomical features is essential in developing effective treatment plans [4]. Overall, CAD/CAM software is an efficient tool that provides medical professionals with the means to improve patient outcomes through accurate and detailed anatomical representations. While stereolithography represents a remarkable breakthrough in imaging innovation, certain limitations persist, such as precision of detail and cost considerations associated with producing stereolithographic models.

Overall, stereolithography and CAD/CAM software have emerged as indispensable tools in the medical field, significantly improving diagnostic accuracy and treatment quality when coupled with meticulous surgical planning and execution.

The objective of this investigation was to evaluate the efficacy of stereolithographic models in diagnosing and planning maxillofacial surgeries through direct visualization and simulated surgical procedures on the models to enhance surgical precision and postoperative outcomes. Utilizing helical volume CT scans of the relevant anatomical regions, necessary data were obtained for the construction of these models. Rather than relying on quantifiable variables, the study assessed its success through several criteria: firstly, by measuring the duration of the surgical procedure from incision to the placement of the final suture in hours and minutes; secondly, by comparing the surgical outcomes achieved with those anticipated from preoperative model surgeries conducted on rapid prototype models; and finally, by evaluating the utility and structural fidelity of the rapid prototype models using a specialized scoring system developed by Gillespie et al. [5].

## Case presentation

This case series presents the management outcomes of five patients with complex maxillofacial deformities treated at the Department of Oral and Maxillofacial Surgery, Sri Ramaswamy Memorial (SRM) Dental College, Ramapuram, Chennai. Each patient underwent thorough clinical examinations and radiographic assessments, including orthopantomogram (OPG), lateral cephalogram, and frontal cephalogram. Stereolithographic models were crafted using Materialize software, designed to recognize DICOM-formatted CT scans with a slice thickness of 0.5 mm. These scans were then processed by a rapid tooling machine, which sliced the model into layers, typically 0.5 mm thick. The machine utilized this sliced data to construct the model layer by layer, with each layer seamlessly bonded to the previous one, resulting in a stepped appearance.

Case 1

**Figure 1:**
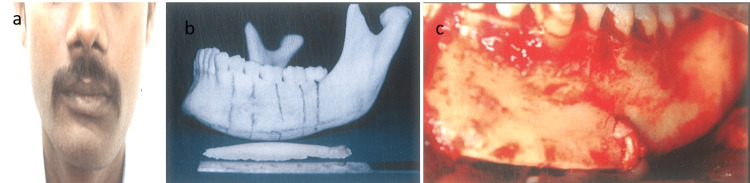
(a) Preoperative frontal view. (b) Comparison of the amount of bone resected with the stereolithographic model. (c) View after completion of the procedure

A 25-year-old male presented with facial asymmetry to our department. Grummons analysis, based on a posteroanterior (PA) cephalogram, indicated mandibular asymmetry attributed to condylar hyperplasia. Subsequent CT scans with 3D reconstruction confirmed the diagnosis, revealing asymmetry predominantly on the left side of the mandible. A prototype stereolithographic model was then generated to visualize the anatomical irregularities. The surgical strategy entailed a left-sided mandibular inferior border body ostectomy. Prior to surgery, a preoperative acrylic surgical template, mirrored from the unaffected right side, was meticulously crafted on the model to guide the procedure. Under general anesthesia, an intraoral sub-vestibular incision was made extending from the 31 to 37 regions, allowing for optimal exposure of the mandibular inferior border (Figure 1a-1c). The template was accurately positioned onto the surgical site, facilitating precise delineation of excess bone through bur hole markings along the lower border. Subsequently, a meticulous lower border body ostectomy was executed, followed by the closure of the mucoperiosteal flap using 4-0 Vicryl sutures.

Case 2

**Figure 2:**
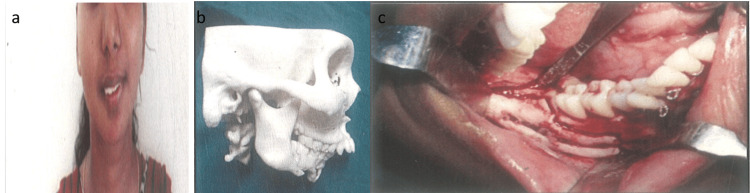
(a) Preoperative frontal view. (b and c) Preoperative lateral view of stereolithographic model

A 26-year-old female patient presented with facial asymmetry, prompting Grummons analysis which confirmed mandibular asymmetry. Radiographic examination unveiled a history of left condylectomy and subsequent postsurgical deformity on the right mandibular body. A prototype stereolithographic model was crafted to aid in treatment planning. The proposed surgical approach involved reconstructing the right inferior border of the mandibular body. A pre-bending technique was employed using a 6×4 cm titanium mesh, precisely adapted to the prototype model. This pre-bent mesh was intended to serve as a template during surgery for the creation of a mandibular body tent, facilitating the receipt of an iliac cortico-cancellous bone graft. Under general anesthesia, the pre-bent titanium mesh was accurately positioned along the lower border of the mandible. Subsequently, an anterior approach was utilized to harvest a right iliac cortico-cancellous bone graft, which was then carefully filled into the mesh and secured in place using 2x8 mm screws (Figure 2a-2c).

Case 3

**Figure 3:**
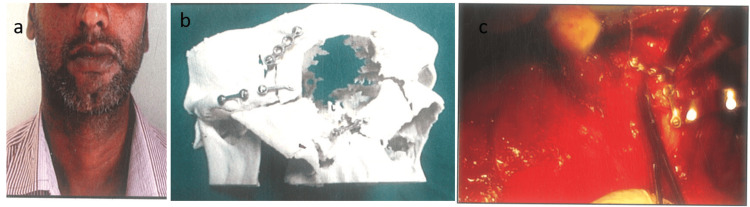
(a) Preoperative frontal view. (b) 1.5 mm two holes with gap miniplate fixed over the zygomatic arch and 1.5 mm four holes with gap miniplate fixed over the infraorbital region. (c) Semi-rigid fixation done intraoperatively of the zygomatico-orbital complex with pre-adapted miniplates

A 32-year-old male presented to our department with a complaint of post-traumatic deformity on the right side of his face. Clinical examination revealed notable irregularities including a step on the right fronto-zygomatic suture, zygomatic body, and lateral wall of the nose, accompanied by increased scleral show and enophthalmos. However, there was no associated visual impairment. Radiographic assessment confirmed a post-traumatic surgical deformity of the zygomatico-orbital complex. Subsequent CT scans with 3D reconstruction facilitated the creation of a prototype stereolithographic model. Based on this model, a surgical plan was devised to address the malunited zygomatico-orbital complex fragment through refracturing and semi-rigid fixation with miniplates. This preoperative planning was meticulously replicated during surgery conducted under general anesthesia (Figure 3a-3c).

Case 4

**Figure 4:**
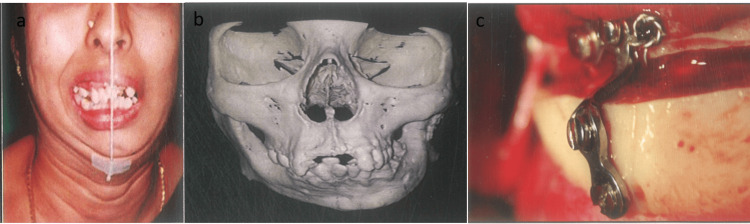
(a) Preoperative frontal view. (b) Preoperative frontal view of the stereolithographic model. (c) L plate fixed intraoperatively after advancement extended sliding genioplasty

A 29-year-old female presented with facial asymmetry attributed to a unilateral post-ankylotic surgical deformity on the left side. Utilizing a prototype stereolithographic model, preoperative planning was conducted to replicate the surgical procedure. The chosen approach involved an advancement extended sliding genioplasty to correct the asymmetry as a camouflage technique. Under general anesthesia, a vestibular incision was made from tooth 36 to tooth 46, and a mucoperiosteal flap was raised. Careful protection of the mental nerve was ensured during the osteotomy cut placed from tooth 36 to tooth 46. The osteotomy segment was then advanced and rotated as planned in the model surgery and secured with an L plate along the anterior border of the mandible (Figure 4a-4c).

Case 5

**Figure 5:**
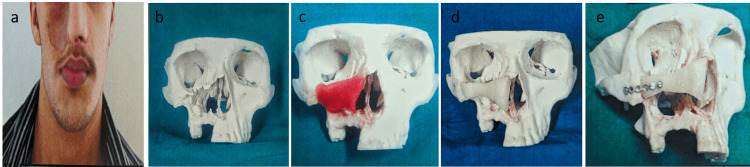
(a) Preoperative frontal view. (b) Preoperative frontal view of the stereolithographic model showing right-side maxillary defect. (c) Preoperative wax-up done over the defect. (d) Acrylic template fabricated over the wax pattern. (e) Acrylic template fixed with 1.5 mm six holes without gap over the zygomatic buttress

A 21-year-old male presented with a post-surgical midface defect on the right side extending from the lower border of the orbit to the upper lip, anteriorly to the lateral wall of the nose, and posteriorly to the zygomatico-maxillary buttress. Previous history revealed surgical correction for odontogenic keratocyst on the right side of the maxilla one year prior. Radiographs and CT scans with 3D reconstruction facilitated surgical planning for the reconstruction of the right maxilla (Figure 5a-5e).

**Figure 6:**
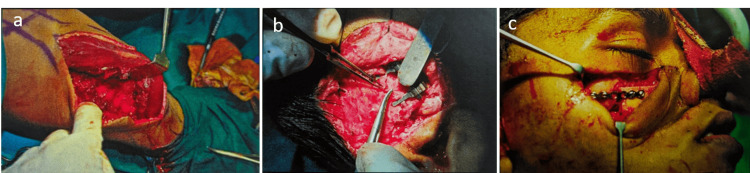
(a) Free fibular bone graft harvested by placing the pre-fabricated acrylic template over the donor site to obtain the same size and shape for the graft. (b) Pedicle with peroneal artery and vein anastomosed with superficial temporal artery. (c) Stainless steel six-hole straight plate and screws placed for the fixation of the graft

A stereolithographic model was fabricated to simulate the defect, and a preoperative wax-up was performed for the right maxilla to create an acrylic template for the bone graft. The template was utilized to assess the size and shape of the bone graft at the donor site. A microvascular free fibular bone graft was harvested for reconstruction via both extraoral and intraoral approaches. Anastomosis of the pedicle with the peroneal artery and vein to the superficial temporal artery and vein was performed as end-to-side anastomoses. Fixation of the graft was achieved using a stainless steel six-hole straight plate and screws (Figure 6a-6c).

Follow-up evaluations were conducted for all five cases, with postoperative assessment based on surgical duration and accuracy compared to model surgery. Additionally, the models' utility in identifying structural details and the quality of visual information were analyzed using the special scoring system by Gillespie et al. (Table 1). The results were tabulated in the succeeding tables for comprehensive analysis [5].

**Table 1:** Scoring system according to Gillespie et al. Attribution and permission were obtained for the utilization of this table Reference: [5]

Score	Description of the quality of visual information
1	Inferior
2	Similar/equivalent
3	Superior (similar information more rapidly assimilated)
4	Superior (additional information provided)

All the patients were informed about the diagnosis, and the treatment plan and surgeries were performed under general anesthesia. The detail of each surgery is given in detail in Table 2.

**Table 2:** All the parameters (treatment plan, pre-treatment, and surgery) assessed in the study 3D: three-dimensional; GA: general anesthesia

Patient	Age (years)/sex	Diagnosis	Diagnostic tool	Treatment plan	Workup done (pre-treatment)	Surgery performed
Case 1	25/M	Condylar hyperplasia (left side)	3D CT reconstruction with rapid prototyping	Left-side mandibular inferior border body osteotomy	A preoperative acrylic surgical template was made for lower body osteotomy and mental nerve preservation	Under GA, inferior border body osteotomy was done on the left side using the surgical template
Case 2	26/F	Post-condylectomy mandibular asymmetry	3D CT reconstruction with rapid prototyping	Reconstruction of the right inferior border of the body of the mandible with a titanium mesh and cortico-cancellous graft fills	Preoperative adaptation of titanium mesh (6x4 cm) and pre-surgical bending of the mesh in the form of tent	Under GA, pre-bent titanium mesh was fitted into the right lower border of the body of the mandible. Cortico-cancellous bone from the iliac crest was taken and filled
Case 3	32/M	Post-traumatic deformity of right-side zygomatico-orbital complex	3D CT reconstruction with rapid prototyping	Refracturing malunited zygomatico-orbital complex fragments and fixed with miniplates	Refracturing done in the model and fixed with miniplates	Refracturing of fronto-zygomatic suture, zygomatic arch, and infraorbital rim was done and semi-rigid fixation was done with pre-adapted miniplates
Case 4	29/F	Unilateral post-ankylotic surgical deformity	3D CT reconstruction with rapid prototyping	Advancement extended sliding genioplasty for asymmetry correction	Advancement extended sliding genioplasty done in the model and fixed with L plate four holes with gap	Under GA, an osteotomy cut was placed from 36 to 46, and the osteotomy segment was advanced and rotated as planned in the model surgery and fixed with an L plate in the anterior border of the mandible
Case 5	21/M	Right-side post-odontogenic keratocyst surgical midface defect	3D CT reconstruction with rapid prototyping	Reconstruction of right-side maxilla using a microvascular free fibular bone graft	Preoperative wax-up done in the model for the right maxilla which was used to fabricate an acrylic template for the bone graft	Under GA, a free fibular bone graft was harvested by placing the pre-fabricated acrylic template over the donor site to obtain the same size and shape for the graft. The pedicle with peroneal artery and peroneal vein was anastomosed with the superficial temporal artery and vein as end-to-side anastomoses. Stainless steel six-hole straight plate and screws were placed for the fixation of the graft

The surgical time was calculated from the start of the incision to the placement of the last suture for closure. As we performed the model surgery, we reduced the time utilization for identifying the pathology or the deformity and its correction, plate adaptation, and fixation. The details of the surgical time were tabulated for each patient (Table 3). The time consumed for performing the surgery ranged from two to three hours. However, for one patient, it took 11 hours as it was the longest surgical procedure.

**Table 3:** The details of the operating time of all the patients as well as the score given for each case based on Gillespie et al.'s scoring system for the description of the quality of visual information

Patient	Age (years)/sex	Diagnosis	Surgery achieved as per model surgery	Scoring	Operating time
Case 1	25/M	Mandibular hyperplasia (left side)	Achieved as per model surgery	3	2 hours
Case 2	26/F	Post-condylectomy mandibular asymmetry	Achieved as per model surgery	2	2 hours 30 minutes
Case 3	32/M	Post-traumatic deformity of right-side zygomatico-orbital complex	Achieved as per model surgery	2	3 hours
Case 4	29/F	Unilateral post-ankylotic surgical deformity	Achieved as per model surgery	3	1 hour 30 minutes
Case 5	21/M	Right-side post-odontogenic keratocyst surgical midface defect	Achieved as per model surgery	3	11 hours

The scoring was given based on the structural details of the rapid prototyping models. In cases 1, 4, and 5, the score was given 3 as the structural details were superior and it was similar information which was visualized intraoperatively (Table 3). In cases 2 and 3, the score was given 2 as the structural details were almost similar-equivalent when compared intraoperatively. Some minor information could not be retrieved in the orbital floor due to its discontinuity in the bones in case 3 and the details of the lower border of the body of the mandible and its continuity in case 2 (Table 3). The results that we achieved were satisfactory as the model surgery reduced the operative time for all the cases.

## Discussion

The emergence of rapid prototyping, utilizing techniques such as stereolithography and 3D reconstruction, has revolutionized various applications within maxillofacial surgery. Particularly, in cases of surgical resection, restoring both the premorbid shape and function stands as a primary objective [6]. Our study leveraged 3D biomodels to diagnose conditions accurately and forecast treatment outcomes with precision.

The cases presented demonstrate the efficacy of utilizing pre-contoured miniplates, crafted through stereolithography, in addressing post-traumatic and other maxillofacial defects. This innovative approach significantly reduced operative time by approximately 1.5 hours per case. Moreover, the pre-bent plates facilitated easier refracturing both intraorally and extraorally, enabling a more conservative surgical strategy. The integration of 3D models into preoperative planning provided surgeons with tangible representations of patients' anatomies, streamlining decision-making processes. In one instance, a malunion and post-traumatic deformity of the zygomatico-orbital complex fracture were successfully treated through refracturing, precise reduction, and fixation using adapted miniplates. This approach not only yielded favorable outcomes but also minimized surgical duration. Similarly, in a case involving deformity of the right lower border of the mandible, a titanium mesh was custom-adapted to match the contour of the defect based on the model. Cortico-cancellous bone grafts, harvested during the procedure, were then placed within the mesh for augmentation. This tailored approach ensured optimal fit and function, resulting in enhanced patient outcomes [7,8].

While possessing a model of the distorted anatomy may offer valuable insights, these models must be manipulated to achieve symmetry. This is generally achieved by contouring the model using a surgical bur, saw, or other grinding tools. The precision and accuracy of these models are of utmost importance, as it allows for meticulous preoperative planning and modifications of the reconstruction plates [9]. A case which was included in our study was diagnosed to be mandibular asymmetry and planned for lower border ostectomy. The model surgery was done in the 3D model, and a template was created using the other side of the mandible to get the symmetry. The lower border was osteotomised using bur and removed along with mental nerve protection. The same was carried out in the surgery which had a precise result and reduction in the surgical time.

One of the primary drawbacks associated with stereolithographic models lies in the significant time and cost investment required for their fabrication. Although advancements in stereolithography technology and materials hold promise for reducing these expenses, the fabrication time remains a potential barrier, particularly in time-sensitive scenarios such as trauma surgery guidance. Moreover, there are concerns regarding potential radiation exposure for patients due to the requisite CT scans necessary for generating the digital data used in stereolithography. Additionally, depending on the type of resin utilized, there may be instances of shrinkage during the building process and even after curing, posing further challenges in maintaining dimensional accuracy [10].

The template serves as the crucial link bridging the gap between planning and execution within a treatment strategy. Consequently, it's imperative that the plan is rooted in a comprehensive comprehension of bone anatomy vis-à-vis the patient's restorative requirements, thereby eliminating any guesswork from the equation. The definitive simulation can then be transformed into a meticulously crafted surgical template, ensuring a precise and successful treatment outcome [11].

Cunningham et al. [11] conducted a mandibular reconstruction following tumor excision, while Perry et al. explored orbital reconstruction in individuals with facial atrophy, fibrous dysplasia impacting the orbit, and tumors affecting the orbital region. Several other researchers concur that the utilization of biomodels facilitates preoperative planning and adjustments to reconstruction plates. In our study, one of the patients underwent maxillary reconstruction utilizing a free fibular graft. Through preoperative planning and model surgery, we were able to efficiently harvest the graft with precise size and shape, greatly enhancing the procedural ease and accuracy.

Sykes et al. [12] conducted an assessment of the prototype's accuracy by replicating non-affected anatomical structures through prototyping and comparing them to those obtained via silicone impression. The researchers highlighted rapid prototyping's primary advantage as the attainment of highly accurate prostheses. Similarly, Ono, Sailer, Winder, and Bibb reported relatively high prototype accuracy, with standard errors ranging from 0.1 to 0.6 mm. This precision is contingent upon factors such as the thinness of CT scans, ideally ranging from 1 to 2 mm, and a resolution of 512x512 for the field of view, while ensuring minimal tilting during image acquisition. In our investigation, we maintained a slice thickness of 0.5 mm for CT scans to enhance the accuracy of rapid prototyping model fabrication.

When CT images undergo conversion into STL format, the surface of the CAD model transforms into numerous finely detailed triangles, thereby enhancing image quality and refining the image-reslicing algorithm [9]. For the purpose of enhancing diagnostic precision and treatment planning, computer-generated images can be exported to rapid prototyping and modeling workstations. These workstations, utilizing a CAM system, fabricate solid models, real prototypes, based on virtual models generated within a CAD system. Despite these advancements, the accuracy and specific indications for utilizing such models remain ambiguous [13].

The SLS model exhibited superior reproduction of mandibular dimensions compared to alternative techniques. This heightened accuracy could potentially be attributed to sandblasting, which induces surface wear. Remarkably, this model demonstrated the smallest dimensional error (1.79%) when juxtaposed with the dry mandible. Interestingly, these findings diverge from those reported by Silva et al. [14], who observed a 2.10% error when comparing a dry skull with a stereolithographic model. Additionally, Berry et al. [15] noted a variation of 0.64%, albeit in a comparison between stereolithographic models and 3D CT images. It's worth noting that such comparisons might vary from the criterion standard, as discussed by Waitzman et al. [16].

In our investigation, we noted that the dimensional error in the 3D PolyJet model was 3.14%, which was marginally lower than the error rates reported by other researchers for midface (4%), maxilla (4.3%), and orbit (4.7%) measurements. However, it exceeded the errors found for skull base (2%) and craniofacial measures (2.1%). We attribute this slight enlargement in the PolyJet models to the infiltration of cyanoacrylate during the process, potentially contributing to superficial enlargement. Unlike alternative techniques, PolyJet models necessitate minimal finishing, requiring only a jet of pressurized water to eliminate supporting structures and achieve surface smoothness. Additionally, the photopolymer resin utilized in the PolyJet system grants transparency to the models. While this transparency might hinder the visualization of superficial structures, it significantly enhances the visualization of internal structures, such as the mandibular canal's location and extension, crucial factors in dental implant planning.

## Conclusions

Our study illustrated the use of CT-based stereolithographic rapid prototyping models. These models were used not only for pre-surgical assessment but also for performing model surgeries. Although rapid prototyping has a lot of advantages, it has a few limitations regarding its cost. As it is time-consuming, its use in emergency situations which require immediate surgical intervention such as trauma is not possible. Due to these limitations, mostly elective procedures such as correction of mandibular asymmetry and post-traumatic deformity reconstruction were included. Stereolithography is just a stepping stone towards the future of computer-assisted surgeries and robotic navigational surgeries.
